# MXene/graphene oxide nanocomposites for friction and wear reduction of rough steel surfaces

**DOI:** 10.1038/s41598-023-37844-0

**Published:** 2023-07-08

**Authors:** Ali Zayaan Macknojia, Aditya Ayyagari, Elena Shevchenko, Diana Berman

**Affiliations:** 1 Department of Materials Science and Engineering, University of North Texas, 1155 Union Circle, Denton, TX 76201 USA; 2 Center for Nanoscale Materials, Argonne National Laboratory, 9700 South Cass Avenue, Lemont, IL 60439 USA; 3 Department of Chemistry and James Frank Institute, University of Chicago, 929 E 57th St, Chicago, IL 60637 USA

**Keywords:** Graphene, Structural properties, Two-dimensional materials, Mechanical properties, Design, synthesis and processing

## Abstract

Development of solid lubricant materials that render reliable performance in ambient conditions, are amenable to industrial size and design complexities, and work on engineered surfaces is reported. These coatings are composed of Ti_3_C_2_T_*x*_-Graphene Oxide blends, spray-coated onto bearing steel surfaces. The tribological assessment was carried out in ambient environmental conditions and high contact pressures in a ball-on-disc experimental set-up. The evaluation yielded that the use of Ti_3_C_2_T_*x*_-Graphene-Oxide coatings led to substantial reduction in friction down to 0.065 (at 1 GPa contact pressure and 100 mm/s) in comparison to the uncoated of single-component-coated surfaces, surpassing the state-of-the-art. The coatings also provided excellent protection against wear loss of the substrate and counter-face. The results were explained based on the observations from Raman spectroscopy, scanning electron microscopy, transmission electron microscopy, and nanoindentation measurements. *In operando* formation of a dense, hard and stiff, dangling-bond-saturated tribolayer was observed to be the reason for the sustained lubricity even at high test loads and sliding speeds. This report presents the holistic exploration and correlation of structure-property-processing pertaining to the advancement of solid lubrication science.

## Introduction

Solid lubrication technologies have garnered significant research interest from both fundamental research aspects of materials design^1–7^, and applied front for oil-free^8–11^, non-polluting technologies that meet the demands of electric propulsion^12,13^. Solid lubrication is also of interest to extra-terrestrial applications^14–17^ as demonstrated by the systems and technologies developed for the Mars rovers and international space station components^18^. Commercially successful, robust solid lubrication systems such as diamond-like carbon^19,20^, sputter deposited $MoS_2$^21–24^, or other layered (atomic layer deposition, chemical and physical vapor deposition etc.) have pushed the boundaries of oil-free lubrication, even in harsh environments such as humid and high-temperature applications^25–27^. Although these coatings and materials technologies meet the needs of niche applications, the quest for simpler development techniques that are cost-effective and robust under multifarious test conditions is ongoing.

In the context of layered materials, a new class of material systems, having a chemical formula of $M_{n+1}AX_{n}$ that can be further processed to form MXenes^28–30^ has emerged. MXenes are layered transition metal carbides, nitrides, or carbonitrides, that have shown exceptional physical^31^, mechanical^32–36^, energy, chemical and electrochemical^37–40^, bio-imitating^41–43^, optical^44^ and tribological properties^45–48^. Recent reports explore the dry lubrication properties of MXenes and MXene-based coatings in high TRL examples such as roller bearings, thrust ball bearings under ambient conditions^47,49–54^. Recent publication showed superlubric behavior of MXene-based coatings in dry nitrogen atmosphere, with very low dew-point^55^. Despite these advancements, there is a knowledge gap in realizing pathways enabling high lubricity in ambient/humid conditions, as seen in common place applications. The role of surface and terminal bond passivation is not as consequential when tested in inter atmospheres, as compared to humid conditions. Other equally important variables include microstructural morphosis, and chemical bond changes in tribological contacts, all of which affect the application of MXenes in humid and ambient environment applications. In addition to demonstrating impressive metrics of improved wear-resistance and lubrication, it is perhaps more important to explore and understand the *modus operandi* to pave way for continual advancement. This report presents the results of synthesis of an MXene-based solid lubricant coating to render low friction, excellent wear loss prevention, and prolonged performance in ambient conditions. The development of the lubricant coatings itself is kept simple and scalable. In addition to demonstrating the lubrication properties, the mechanisms were explained using electron microscopy, nanoindentation, and Raman spectroscopy techniques. Correlations between structural, chemical, physical, and mechanical properties are also discussed. This multivariate analysis is believed to offer insights into hitherto unexplored aspects of materials tribology and, specifically, engineered tribolayer formation, and to advance solid lubrication science at large. Such would not only be of interest to fundamental research but also serve as a template for accelerated lubricant materials discovery in the applied domain.

## Methods

Pristine micro-crystalline MXene (Ti_3_C_2_T_*x*_) powder obtained from Nanochemazone (Leduc, Alberta, Canada) was suspended in ethyl-alcohol at 35 g/L concentration, and was mixed with Graphene-Oxide aqueous suspension (5 g/L concentration) obtained from Graphene Laboratories Inc. (Ronkonkoma, New York, USA) in 1:1 v/v to form the suspension containing solids-lubricant materials in 1:3 ratio. The suspension containing the solid lubricant materials was spray coated using pneumatic spray-coater with dry nitrogen at 5 psi line pressure on to a 52100-steel substrate having a hardness of 60 HRc, average surface roughness of R_*a*_ = 30 nm, pre-heated to heated to 80 °C. The temperature was maintained such that the carrier liquid evaporated immediately upon contact with the substrate depositing the solids, simultaneously not altering the starting materials’ morphology or tempering the steel substrate. Scrapes from the substrate were lodged onto the lacy-carbon copper grid and imaged using JEOL 2000F transmission electron microscope. The lubrication performance of the coatings was assessed using Anton Paar tribometer in unidirectional sliding mode at 1 N, 10 N and 20 N sliding against 6 mm diameter hardened 52100 steel ball (corresponding to Hertzian contact pressures of 0.48 GPa, 1.0 GPa and 1.13 GPa respectively). Each tribological (sliding) test was performed at least three times. The total sliding distance was kept constant at 30 m, and a velocity at 100 mm/s. Morphology of as-received materials, and tribopairs were imaged using FEI Quanta 200 scanning electron microscope at 5 kV beam voltage. Raman spectroscopy was performed using inVia Renishaw instruments with blue laser (457 nm). Changes in the hardness and modulus of the coating were quantified using the iNano nanoindenter, from KLA Instruments (Milpitas, California, USA). The indentations were made with a diamond Berkovich tip at 10,000 mN load in a continuous sensing mode. A Filmetrics Profilm3D Optical Profilometer was used to generate the 1D and 3D surface, and used to calculate the ball wear and flat rates. Each wear track and ball radius measurements were repeated five times. VersaSCAN micro-scanning electrochemical workstation with scanning kelvin probe (SKP) module was used for relative work function measurements. A tungsten probe was used as a reference for the SKP measurements.

## Results and discussion

**Figure 1:**
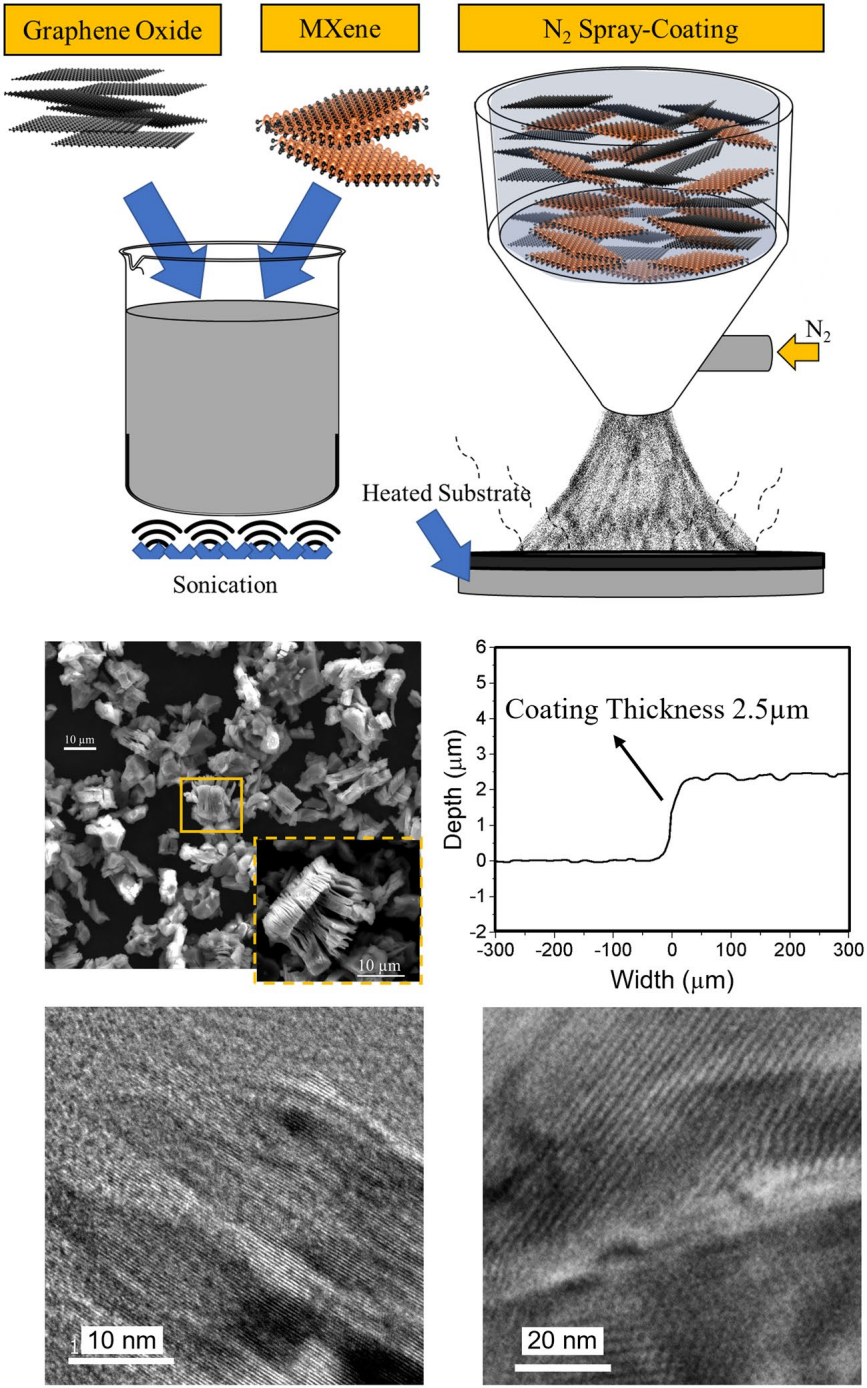
(**a**) Schematic showing synthesis and spray deposition of the lubricants onto the pre-heated steel substrate. (**b**) Scanning electron micrograph showing the morphology of pristine MXenes, inset showing a high magnification image clearly showing accordion-like multi-layer structure (**c**) Coating thickness measured using optical profiler by cutting a step on the substrate. Transmission electron micrographs of (**d**) Graphene Oxide and (**e**) MXene. The lattice parameter calculated from the images is 2.9 Å for Graphene Oxide and 8.2 Å for MXene correspondingly.

Schematics of the suspension preparation and spray deposition processes are shown in Fig. 1a. The suspension was characterized by a very homogeneous distribution (no phase-separation) of MXene and Graphene Oxide in ethanol, with no preferential settlement with time over several-day period. This enabled easy handling, transfer, and holding in the liquid chamber of the pneumatic sprayer-coater. The morphology of the as-received MXene is shown in Fig. 1b, with an average particle size of 7 ± 2 µm and each particle having a distinct accordion-like multi-layer structure. The coating thickness was assessed at multiple locations by cutting through the deposit up to the substrate and measuring the step height using the Filmetrics Profilm3D Optical Profilometer. A representative line profile of the step from the substrate to the top of the coating surface is shown in Fig. 1c. The average coating thickness was observed to be 2.5 ± 0.12 µm. Transmission electron micrographs of Graphene Oxide (shown in Fig. 1d) and of MXene (shown in Fig. 1e) confirm no deleterious changes occurred to the starting materials during synthesis and deposition. The average lattice parameter calculated from the images is 2.9 Å for Graphene Oxide and 8.2 Å for MXene correspondingly, and were found in line with previous reports^7,18,48,50–52,56^.

**Figure 2:**
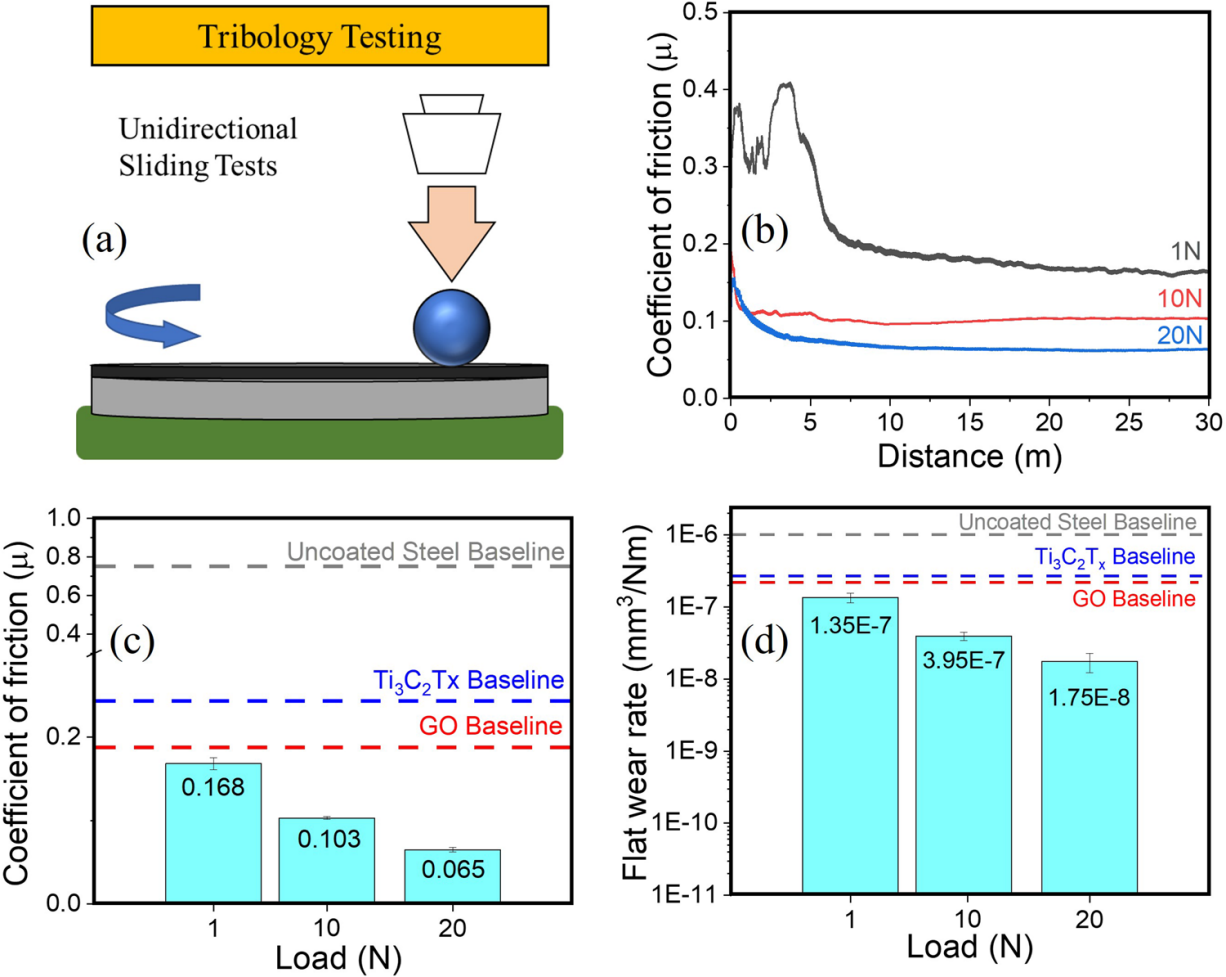
(**a**) Schematic of the pin-on-disc test showing unidirectional sliding test setup (**b**) COF vs sliding distance graphs showing the friction values obtained at 1, 10, and 20 N sliding for 30 m (**c**) histograms showing the average steady-state friction values at the three test conditions (**d**) wear rate on the flat substrate.

A schematic of the tribological experimental setup is shown in Figure 2a. The tests were conducted in unidirectional sliding mode using static weights in ambient conditions (24 $^o$C, 44% RH). The coefficient of friction (COF) vs distance graphs in Fig. 2b show characteristic dry-sliding behavior with a short run-in (also referred to as break-in) period, after which the friction was observed to attain a steady state and remain in equilibrium for the rest of the duration of the experiment. There is a clear trend of decreasing break-in time with increasing normal load, suggesting larger mechanical energy input accelerated the kinetics of materials transformation that effectively resulted in lubricious sliding. The average steady state friction was calculated and plotted as a function of normal load in Fig. 2c. A monotonous decrease in friction was observed as normal load increased, and is consistent with other solid lubricant materials behavior found in literature^7,8,10,22,57^.

The friction for the 1 N test was 0.168, for 10 N was 0.103 and for 20 N was 0.065. These values are significantly lower than those of the bare unlubricated steel-on-steel sliding (by 91.6%), just MXene (by 71%), or just Graphene Oxide (by 64%) even though the later two test coupons were prepared using the same processing conditions and lubricant amount on the surface. While the pristine materials’ lubrication properties are in line with previous reports^51,58,59^, the pronounced decrease in MXene-Graphene Oxide combination, relative to pristine MXene or Graphene Oxide suggests a synergistic mechanism at play that is hitherto not seen with either of the individual components. The summary of wear loss results from the discs measured using white light interferometry are shown in Fig. 2d. Although absolute wear scar diameter increased with increasing normal load, the wear rate, as calculated from Archard’s wear equation decreased monotonically, and was observed to be two orders of magnitude lower than for unlubricated sliding, and at least 90% lower than either of the two individual materials.

**Figure 3:**
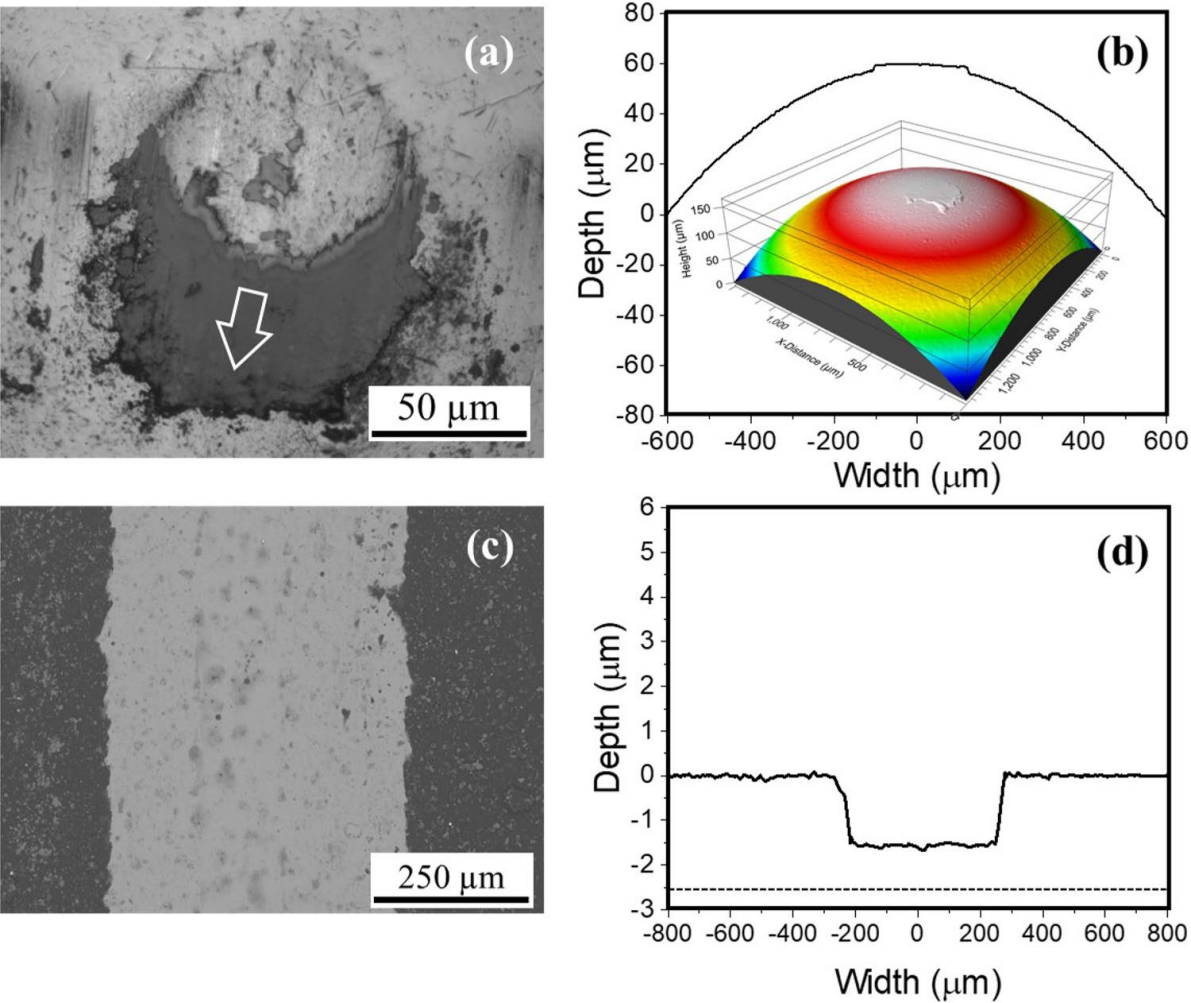
(**a**) Scanning electron micrograph of counterface surface after sliding against the coated flat disc at 20 N for 30 m (**b**) surface profile and three-dimension isometric re-construction of white light interferometry scans of the counterface surface (**c**) surface of the discs with pristine coating appearing darker and sliding path appearing brighter (**d**) surface profile of the coating with depression resulting from sliding under normal load. The scanning electron micrograph of the counterface shows the adhesion of the coating material transferred forming a lubricious tribofilm. The sliding direction is shown on the figure with white arrow. The tribofilm formation can also be seen in the line scan and surface scans. The wear track did not show any rupture or exposure of underlying steel as evidenced by the surface scan data showing about 500 nm of coating underneath the sliding path at the end of the sliding experiments.

The scanning electron microscopy micrograph of the counterface surface is shown in Fig. 3a, and corresponding line (1D) and surface (2D) interferometry scans of the contact regions are shown in Fig. 3b. It is evident from the three inputs that a robust transfer layer is formed on the counterface during sliding. This layer resisted dislodging during commonplace sample wiping, handling/movement between various analyses and storage, suggesting its firm adherence to the counterface. The micrographs and interferometry data also showed no ball flat (or cap) formation, confirming no steel materials loss. The scanning electron micrograph of the wear track formed on the flat side is shown in Fig. 3c with d showing the corresponding line and 3D profiles of the surface. The micrographs did not show any rupture and consequent exposure of underlying steel. The profilometry data showed up to 500 nm of coating material underneath the wear track at the end of the sliding experiments. This tribolayer not only lowered friction, but also prevented wear loss by separating metal-on-metal contact.

**Figure 4:**
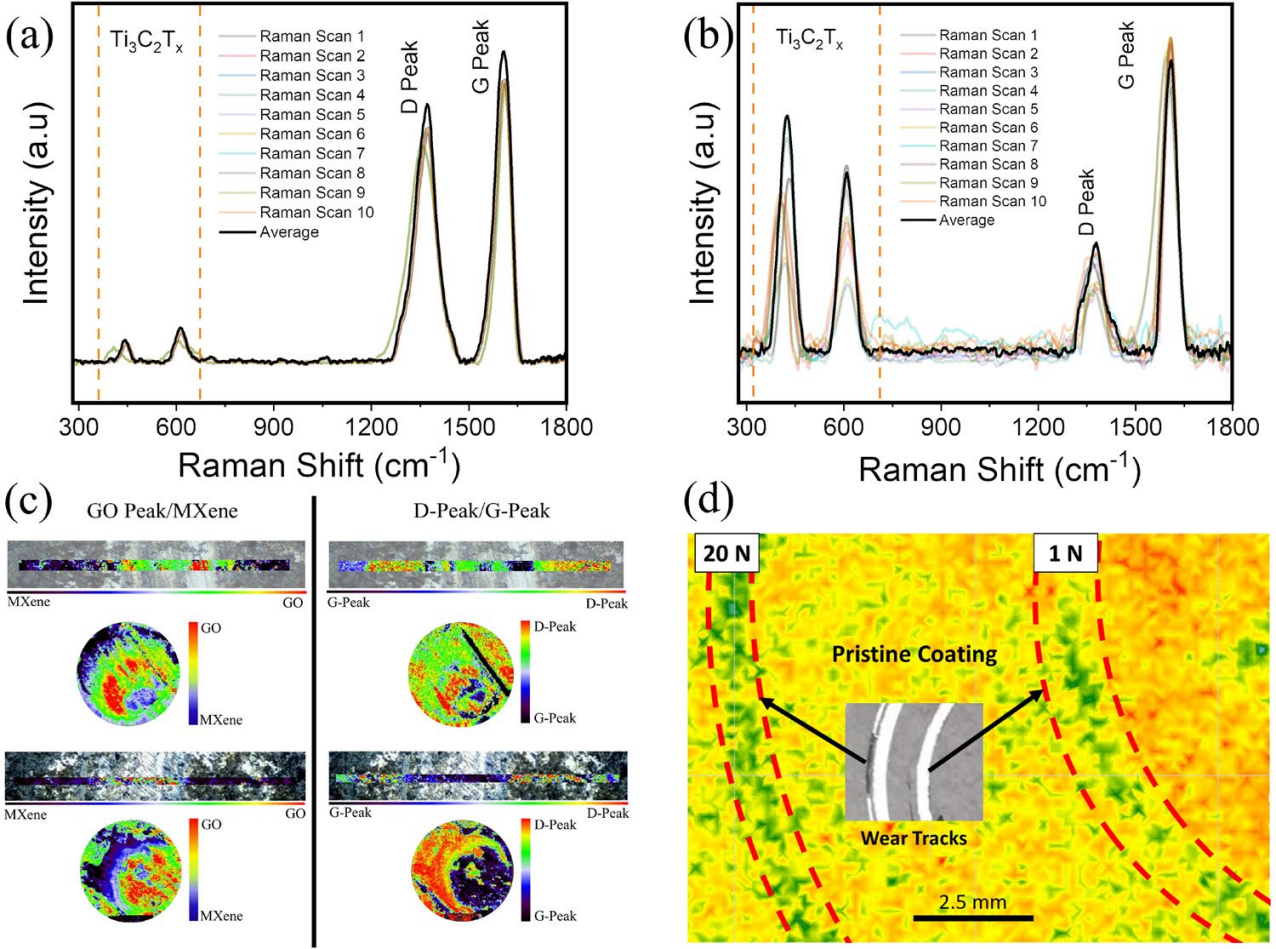
Raman spectrograms showing the multi point scan data and the average from (**a**) 1 N test (**b**) 20 N test, (**c**) surface map and ratios of GO to MXene characteristic peaks. The point and area spectra indicate intensities in the contact area, with a slight prevalence of Graphene Oxide in the center and MXene on the edges at low loads. This trend intensifies at the 20 N test condition. Within the Graphene Oxide spectra, there is an equal prevalence of both G and D peaks at low loads, whereas significant signal strength emanates from the D peak (**d**) Scanning kelvin probe map showing the stiction variation and an in set showing the tracks on the sample.

**Figure 5:**
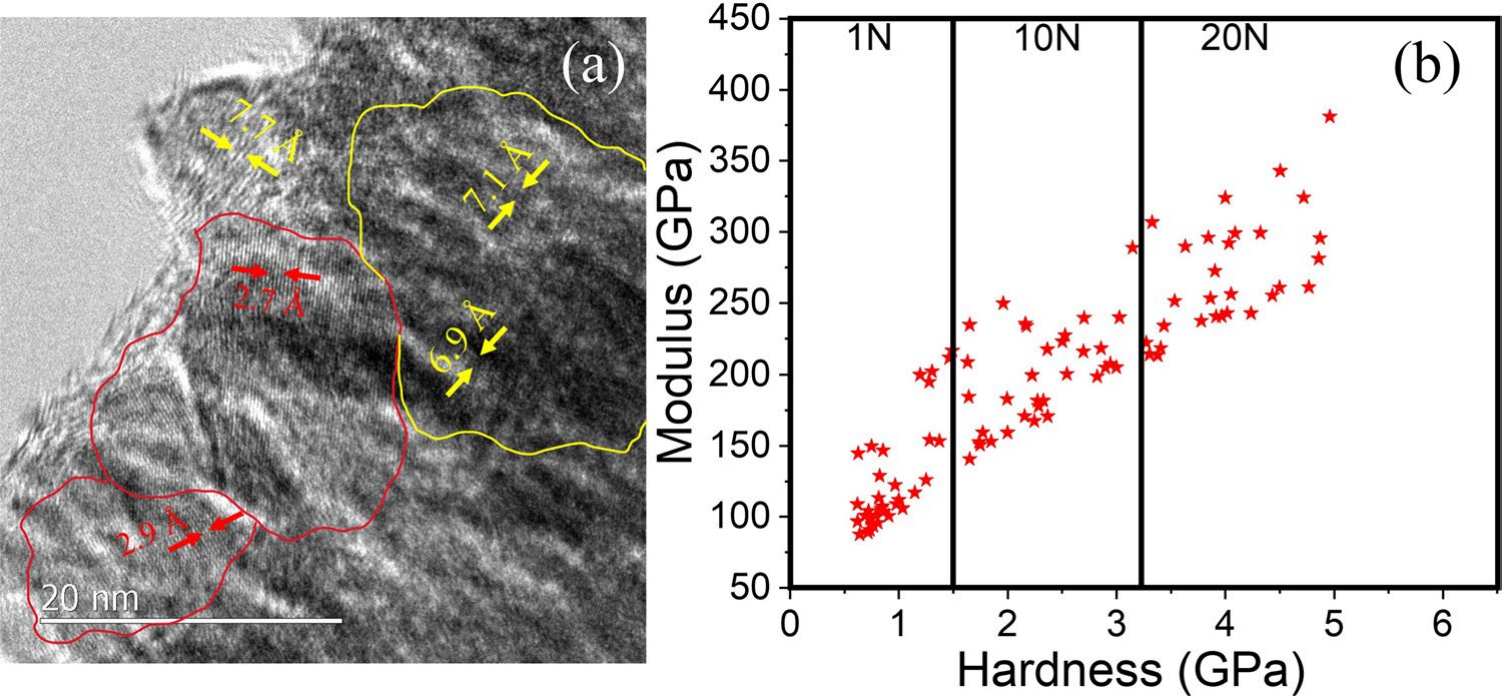
(**a**) Transmission electron mircograph of the sample taken from the tribolayer, showing MXene phase (highlighted with yellow line), and graphitic phase (highlighted with red line) (**b**) Hardness and modules of coating materials measured in the wear track.

The changes in the structure and bonding of lubricant on the counterface (Fig. 3a) and wear track (Fig. 3c), as measured by Raman spectroscopy are shown in Fig. 4. Raman point-scan for the 1 N test condition is shown in Fig. 4a, and for 20 N in Fig. 4b. The results indicate that signatures of the pristine materials were retained with pronounced signature emanating from the phase mixture of MXene and Graphene Oxide for the 1 N tribolayer. This is in stark contrast to the Raman signature from the tribolayer after sliding at 20 N, which showed changes corresponding to materials evolution under increased applied stresses. The relative intensity of the D-peak decreased, G-peak increased, and characteristic peaks of the MXene component were observed to increase in intensity. Spectrograms generated over the entire contact areas (Raman maps) are shown in Fig. 4c. The spectrograms from the 1N test showed more of green regions corresponding to the equimolar presence of Graphene Oxide and MXenes on both the disc and the counterface. For the 20 N tribolayers, the phase distribution showed more of red regions corresponding to Graphene Oxide presence in the center, blue regions corresponding to MXene presence on the edges, and green regions corresponding to equal phase presence inherited from early stages of sliding. The Graphene Oxide peaks were further resolved, and de-convolution indicated that G-peak and D-peak had equal prevalence in the 1 N test conditions and strong G-peak predominance in the 20 N test condition in the transfer layer. While the former set of observations indicates that the as-deposited material was not only loosely packed, exposing different orientations to the probing laser source, but also that the mechanical energy input at 1 N was not sufficient to produce any significant material morphosis. On the other hand, more intense G-peak in the 20 N samples may indicate that the larger energy input into the tribosystem resulted in compaction and basal plane alignment of the Graphene Oxide as well as potentially mechanically-induced reduction in oxygen passivation. This mechanical energy converted the several layers of loosely deposited graphene oxide into compact tribolayer resembling thick multi-layer Graphene, tending towards Graphitization. Despite the tests being carried out in an ambient atmosphere, the sliding path was not characterized by the presence of metal oxides or degradation of graphene oxide flakes, which could be due to the removal of structural water from the sliding path^60,61^. Literature indicates that materials with low relative work function, identified as active, anodic or having unsaturated terminal bonds tend to be electron transfer sites^62–64^ resulting in stiction and adhesion, in contrast to inert or passive phases that by virtue of higher work-function, or, bond saturation resist such interactions, effectively resulting in lower friction during sliding^65–71^. The SKP data after the sliding experiments is shown in Fig. 4d. The areas with high stiction are shown with yellow-orange hues and the ones with lower stiction characteristics are shown in green. The materials in sliding path clearly have lower stiction properties compared to the remaining as-deposited coating. Due to compaction with MXenes, which possess metallic nature, higher hardness, provide structural rigidity, and ensuing graphitization, the tribolayer may have attained greater terminal-bond-saturation relative to as-deposited coatings. The predominant *in operando* formed multi-layer Graphene Oxide or graphitic carbon phase, as seen from the Raman spectrograms are known to have higher electron work function compared to single or few-layer graphene, graphene oxide, or reduced graphene oxide^72,73^, aligning with the observed lowered stiction and better lubricious behavior. The results from Figs. 4 and 2 can be summarized as following: higher sliding loads might have accelerated the transformation of pristine single layer Graphene Oxide to multilayer morphology akin to graphitic carbon, while the MXene formed a support truss that provided structural rigidity. This is reflected as stronger G-peak in Raman and greater passivation-driven lubricity in the COF graphs. The representative transmission electron micrograph of samples extracted from the center of the wear tracks (at the end of 20 N test) presented in Fig. 5a shows regions with intact MXene phase (delineated with yellow, having a lattice parameter 6.9–7.1 Å), densely compacted multi-layer-Graphene/Graphitic Carbon phase (delineated with red lines, having a lattice parameter 2.7–2.9 Å) as direct visual evidence supporting the aforementioned hypothesis.

**Figure 6:**
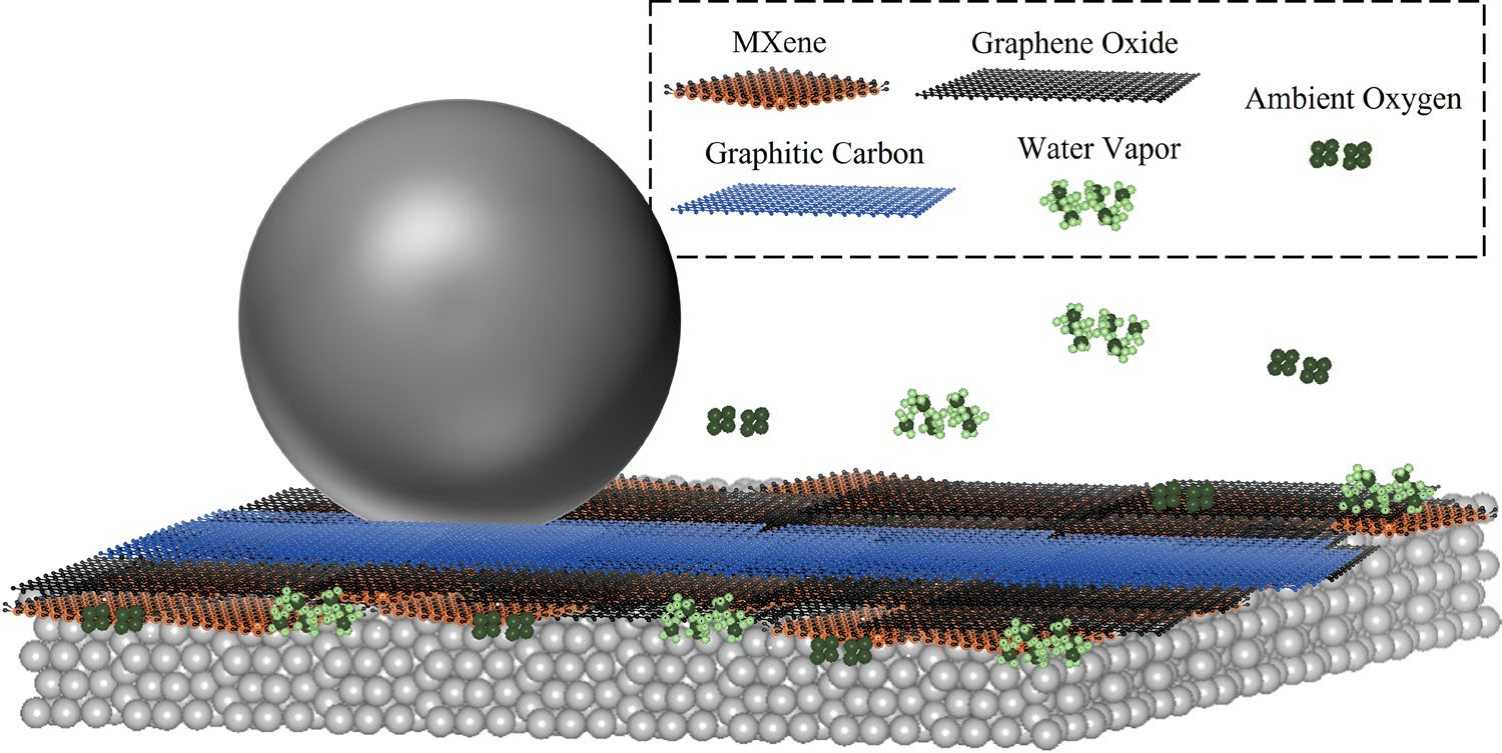
Schematic showing formation of the compacted, hardened and stiff tribolayer composed of graphitic carbon bonded with MXene.

The tribomechanical response measured using nanoindentation is shown in Fig. 5b. The indentation measurements across the three wear tracks show a clear trend of increasing hardness with increasing normal load. The peak hardness and modulus in the tribolayer after 1N test was ${1.3\pm 0.12}$ GPa and ${150 \pm 15}$ GPa; after 10 N test was ${2.5 \pm 0.25}$ GPa, and ${200 \pm 25}$ GPa; and highest values were ${4.5 \pm 0.35}$ GPa and ${300 \pm 22}$ GPa in the wear track after 20 N test. The hardness of the substrate was measured to be ${6.0 \pm 0.2}$ GPa, while the measured hardness values of the tribolayers were well below 5.0 GPa, indicating that substrate effects did not produce any pronounced artifacts in the measured values. Summarily, there emerged a clear trend of compaction of the deposited powder, which resulted in improved hardness and enhanced stiffness during sliding under high contact pressures. These tribomechanical observations in conjunction with remnant coating material after sliding (Fig. 3d), graphitization and saturation of dangling bonds in MXene-Graphene Oxide composite during sliding, indicate *in operando* transformation of materials in the tribolayer that contributed to the sustained lubricity performance. These mechanistic transformations are shown in Fig. 6. .

## Conclusions

This study reported the wear and friction behavior observed for a novel binary composite of Ti_3_C_2_T_*x*_ and Graphene Oxide synthesized using a spray-coating process. The coating was observed to provide excellent wear resistance on the substrate, and no detectable wear loss on the counterface ball. The friction values were significantly lower as compared to the state-of-the-art materials tested in ambient temperature and humidity conditions in contact pressures over 1 GPa. This behavior was explained based on observations from white-light-interferometry, scanning and transmission electron microscopy, Raman spectroscopy (point and areal mapping), and nanoindentation measurements, as the formation of a robust tribolayer that was composed of MXene and graphitic carbon transformed from Graphene Oxide compaction, which prevented metal-on-metal contact. This tribolayer was characterized to possess high hardness, stiffness, and surface passivation that enabled excellent lubrication behavior. The success of this simple coating synthesis advances the technology readiness of solid lubricants for complex and real-world applications.
