# Author Correction: MXene/graphene oxide nanocomposites for friction and wear reduction of rough steel surfaces

**DOI:** 10.1038/s41598-023-44283-4

**Published:** 2023-10-11

**Authors:** Ali Zayaan Macknojia, Aditya Ayyagari, Elena Shevchenko, Diana Berman

**Affiliations:** 1 Department of Materials Science and Engineering, University of North Texas, 1155 Union Circle, Denton, TX 76201 USA; 2 Center for Nanoscale Materials, Argonne National Laboratory, 9700 South Cass Avenue, Lemont, IL 60439 USA; 3 Department of Chemistry and James Frank Institute, University of Chicago, 929 E 57th St, Chicago, IL 60637 USA

Correction to: *Scientific Reports* 10.1038/s41598-023-37844-0, published online 08 July 2023

The original version of this Article contained an error in Figure 1e. Due to a mistake in figure assembly, this panel was inadvertently duplicated from Figure 1c in the work of Macknojia, A. et al.^1^ The original Figure 1 and accompanying legend appear below.

**Figure 1:**
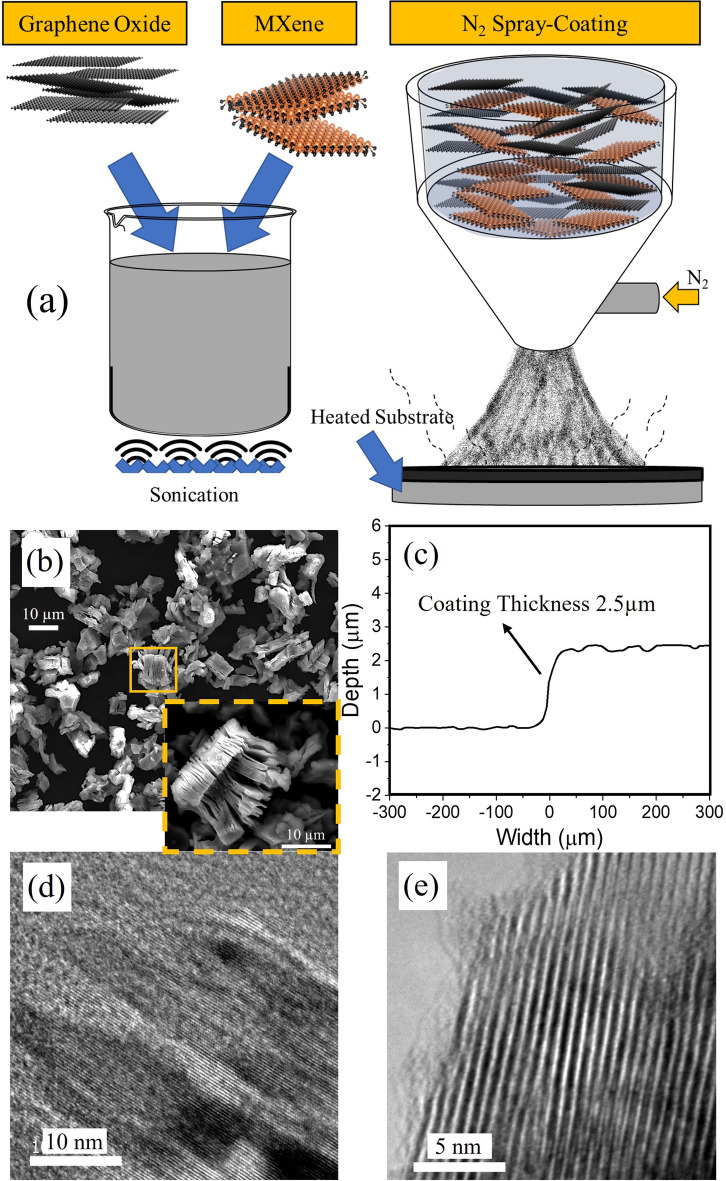
(**a**) Schematic showing synthesis and spray deposition of the lubricants onto the pre-heated steel substrate. (**b**) Scanning electron micrograph showing the morphology of pristine MXenes, inset showing a high magnification image clearly showing accordion-like multi-layer structure (**c**) Coating thickness measured using optical profiler by cutting a step on the substrate. Transmission electron micrographs of (**d**) Graphene Oxide and (**e**) MXene. The lattice parameter calculated from the images is 2.9 Å for Graphene Oxide and 8.2 Å for MXene correspondingly.

The original Article has been corrected.
